# Supplementary figures and images for: A duplex real-time PCR with probe for simultaneous detection of Geosmithia morbida and its vector Pityophthorus juglandis
Source: PLoS One. 2020 Oct 23;15(10):e0241109. doi: 10.1371/journal.pone.0241109 (PMC7584228; doi:10.1371/journal.pone.0241109)

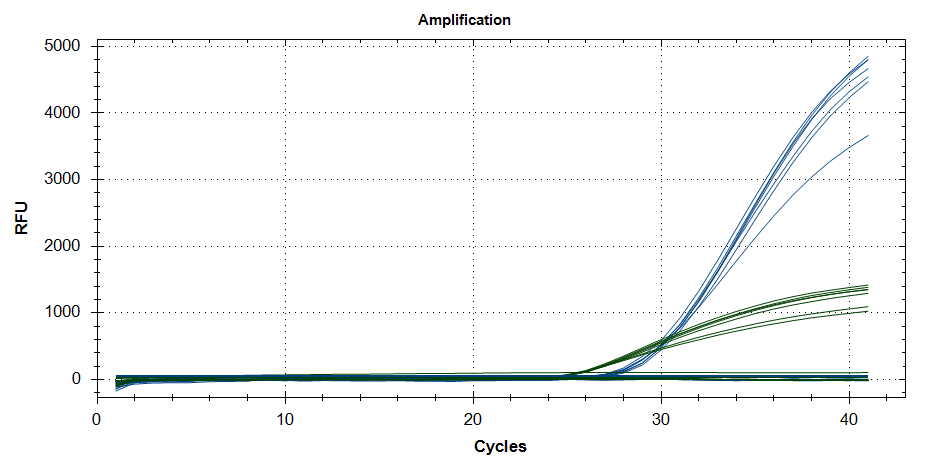

Supplement: S1 Fig — Curves represent different samples for each target. Curves represent different samples for each target. (TIF) [file pone.0241109.s001.tif]
