# Supplementary material for: A duplex real-time PCR with probe for simultaneous detection of Geosmithia morbida and its vector Pityophthorus juglandis
Source: PLoS One. 2020 Oct 23;15(10):e0241109. doi: 10.1371/journal.pone.0241109 (PMC7584228; doi:10.1371/journal.pone.0241109)
Supplement: S2 Fig — (PPTX) [file pone.0241109.s002.pptx]

## Slide 1
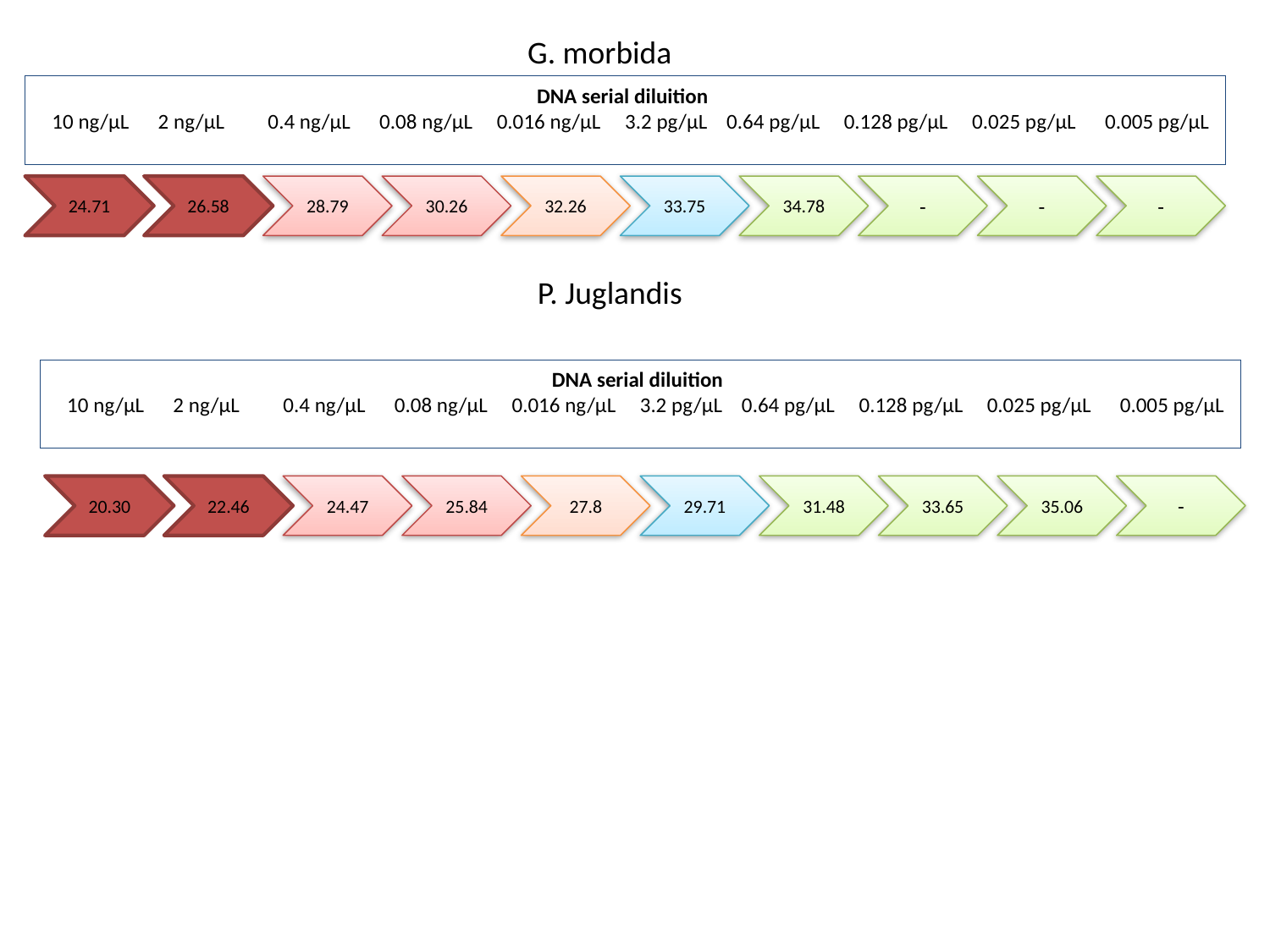

G. morbida
DNA serial diluition
 10 ng/µL 2 ng/µL 0.4 ng/µL 0.08 ng/µL 0.016 ng/µL 3.2 pg/µL 0.64 pg/µL 0.128 pg/µL 0.025 pg/µL 0.005 pg/µL
24.71
26.58
28.79
30.26
32.26
33.75
34.78
-
-
-
P. Juglandis
DNA serial diluition
 10 ng/µL 2 ng/µL 0.4 ng/µL 0.08 ng/µL 0.016 ng/µL 3.2 pg/µL 0.64 pg/µL 0.128 pg/µL 0.025 pg/µL 0.005 pg/µL
20.30
22.46
24.47
25.84
27.8
29.71
31.48
33.65
35.06
-
