# Supplementary material for: A duplex real-time PCR with probe for simultaneous detection of Geosmithia morbida and its vector Pityophthorus juglandis
Source: PLoS One. 2020 Oct 23;15(10):e0241109. doi: 10.1371/journal.pone.0241109 (PMC7584228; doi:10.1371/journal.pone.0241109)
Supplement: Attachment — Submitted filename: Response to Reviewers.docx [file pone.0241109.s004.docx]

Firenze, 07-October-2020

Dear Reviewers,

Thank you for your valuable comments on our manuscript entitled " **A duplex real-time PCR with probe for simultaneous detection of *Geosmithia morbida* and its vector *Pityophthorus juglandis*”**, by Domenico Rizzo, Daniele Da Lio, Linda Bartolini, Giovanni Cappellini, Tommaso Bruscoli, Matteo Bracalini, Alessandra Benigno, Chiara Salemi, Dalia Del Nista, Antonio Aronadio, Tiziana Panzavolta and Salvatore Moricca.

We appreciated your comments, which helped us to improve our manuscript and make it to appear in a better light. We have made all changes you suggested and we trust that you will find them in order and appropriate. Please do not hesitate to contact us if there are any questions regarding the changes made.

Best regards,

Tiziana Panzavolta
